# Supplementary material for: Growth and Thermo-driven Crystalline Phase Transition of Metastable Monolayer 1T′-WSe2 Thin Film
Source: Sci Rep. 2019 Feb 25;9:2685. doi: 10.1038/s41598-019-39238-7 (PMC6389884; doi:10.1038/s41598-019-39238-7)
Supplement: Supplementary file 1 — Supplementary Information [file 41598_2019_39238_MOESM1_ESM.pdf]

Supplementary Informaion for

Growth and Thermo-driven Crystalline Phase Transition of Metastable Monolayer 1T’-WSe_2 Thin Film

Wang Chen, Xuedong Xie, Junyu Zong, Tong Chen, Dongjin Lin, Fan Yu, Shaoen Jin, Lingjie Zhou, Jingyi Zou, Jian Sun, Xiaoxiang Xi, Yi Zhang*

*Email: zhangyi@nju.edu.cn

A: Ratio of 1T’ to 2H phase of monolayer WSe_2 grown on BLG

We grew some WSe_2 sample on BLG with coverage ~0.4 ML and substrate temperature about 250°C. Then we obtained numerous of STM images. The 1T’ and 2H domains can be easily distinguished by using negative tip bias of -1.5V, which is in the energy gap of 2H-WSe_2 and make it be almost transparent in STM images. We measure the area of the 1T’ domains and 2H domains of

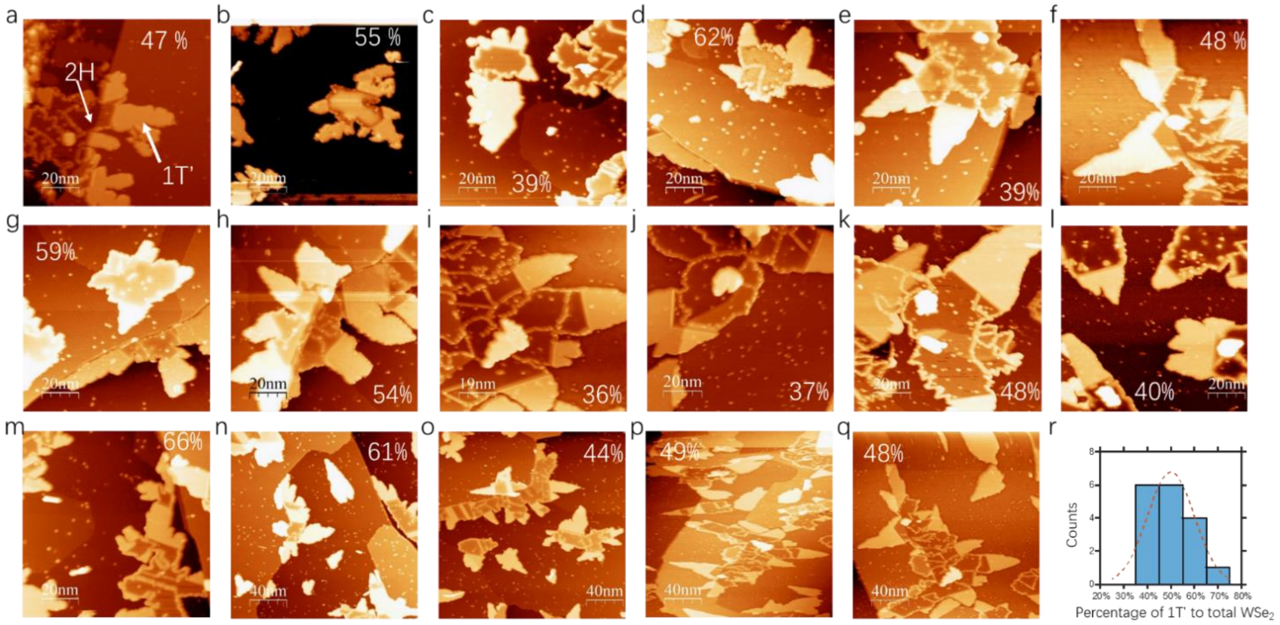

Figure S1. (a)-(q) STM images of WSe_2 film grown on BLG at substrate temperature of 250 °C. The inset percentiles are the percentage of 1T’ phase to the total WSe_2 area in each image. **(r)** Histogram of the percentiles in each image.

each STM images as shown in Figure S1. Then we got the ratio of 1T' to 2H phase is about 1:1.

B: 1T' to 2H phase transition of monolayer WSe_2 grown on BLG

We prepared a WSe_2 on BLG with substrate temperature of ~250ºC and coverage of ~0.3 ML. This low coverage ensure that the monolayer 1T'-WSe_2 only formed on the BLG substrate, no 1T'-WSe_2 islands formed on the top of monolayer WSe_2 layer. Then we did the 400ºC post-annealing. Both the STM images and RHEED patterns show that all the 1T' domains transit into 2H phase (Figure S2). Thus we conclude that for the 1T'-WSe_2 monolayer grown on BLG, the 1T' to 2H phase transition temperature is below 400ºC, which is lower than the phase transition temperature of 1T'-WSe_2 grown on 2H phase that was discussed in main text.

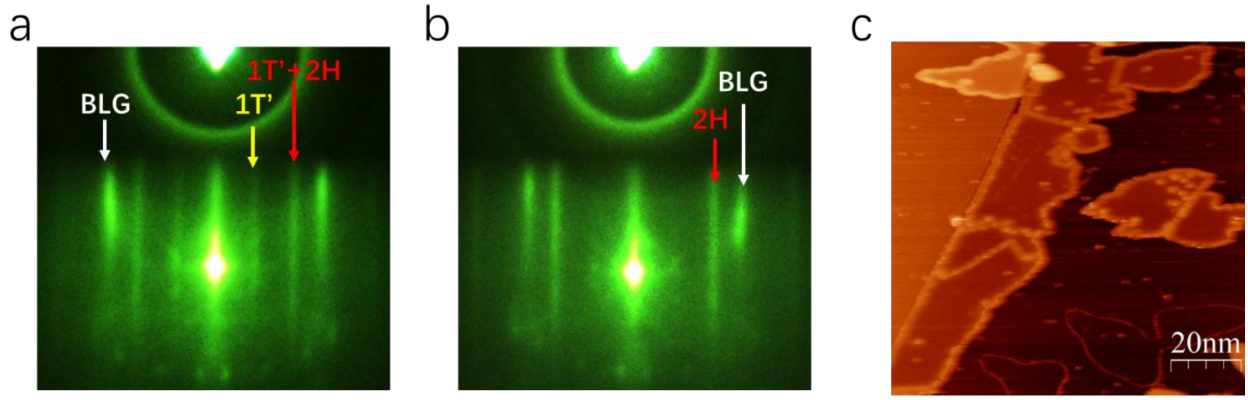

Figure S2. (a) RHEED pattern of a sample with monolayer 1T'-WSe_2 only on the BLG substrate. **(b)** After post-annealing the sample at 400ºC for 20 min, the diffraction spots of 1T' phase was totally disappeared. **(b)** STM image show that all the 1T' phase transited into 2H phase after the post-annealing

C: The duration time for post-annealing procedures

We prepared a sample with ~0.3 ML coverage of 1T'-WSe_2, which have monolayer 1T' domains only grown on BLG but not on 2H-WSe_2 layers (Figure S3a). We first did the post-annealing at 350 ºC for 1 hour, this temperature is below the phase transition temperature. We monitored the

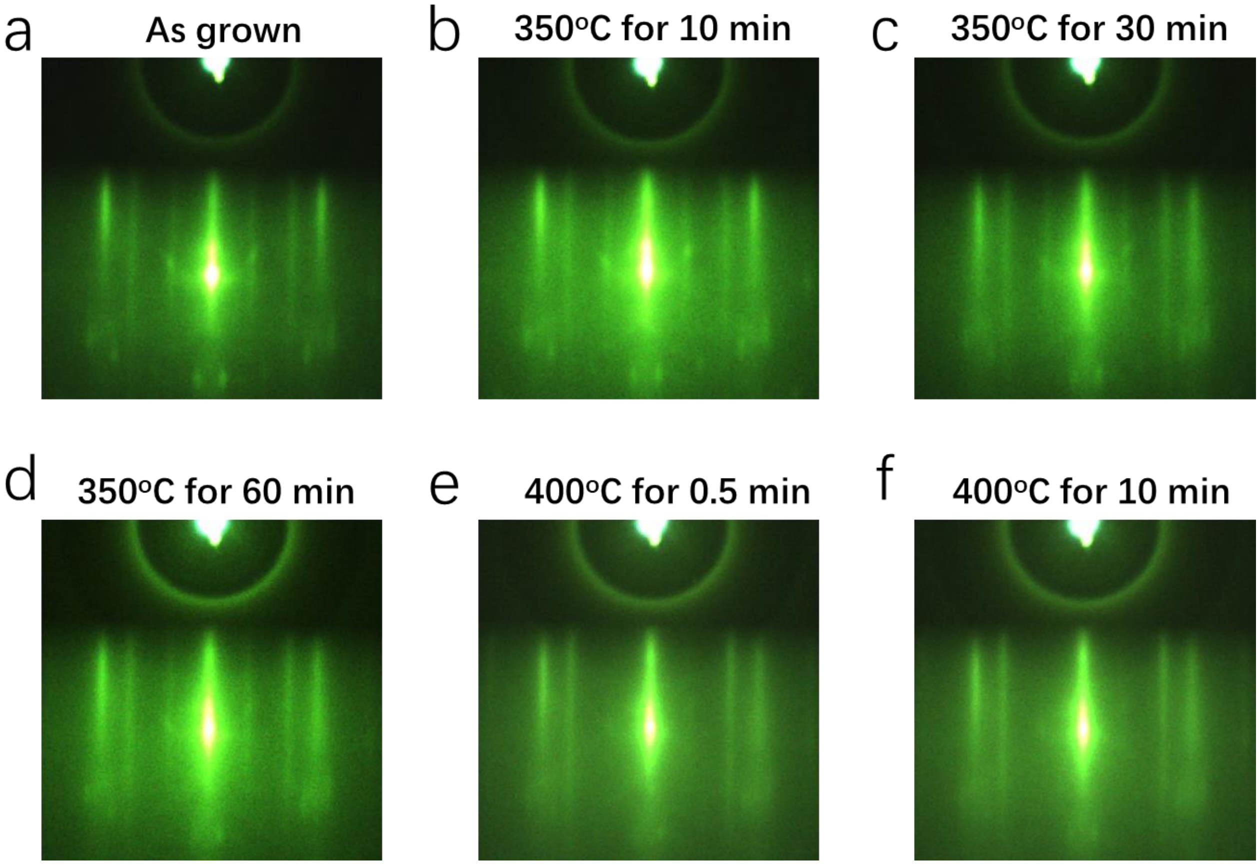

Figure S3. RHEED patterns of a sample with monolayer 1T'-WSe_2 only on the BLG substrate: **(a)** RHEED pattern just taken after the growth. **(b)-(f)** RHEED pattern after different post-annealing procedures.

RHEED image and found that the intensity of diffraction spots from 1T' phase did not decay with the time for post-annealing (Figure S3b-d). Once we increase the annealing temperature up to 400°C, the 1T' to 2H phase transition immediately happens with in few seconds (Figure S3 e&f). Thus we conclude that the 20 minutes post-annealing time mentioned in the main text is longer enough for the totally 1T' to 2H phase transition of WSe_2 monolayer.

D: Different Se flux used for the growth of thin films

We did the WSe_2 growth with different Se fluxes. The temperatures of Se source are ranging from 130°C to 160°C. The corresponding fluxes are from ~1 ML/min to ~10 ML/min. Since the substrate

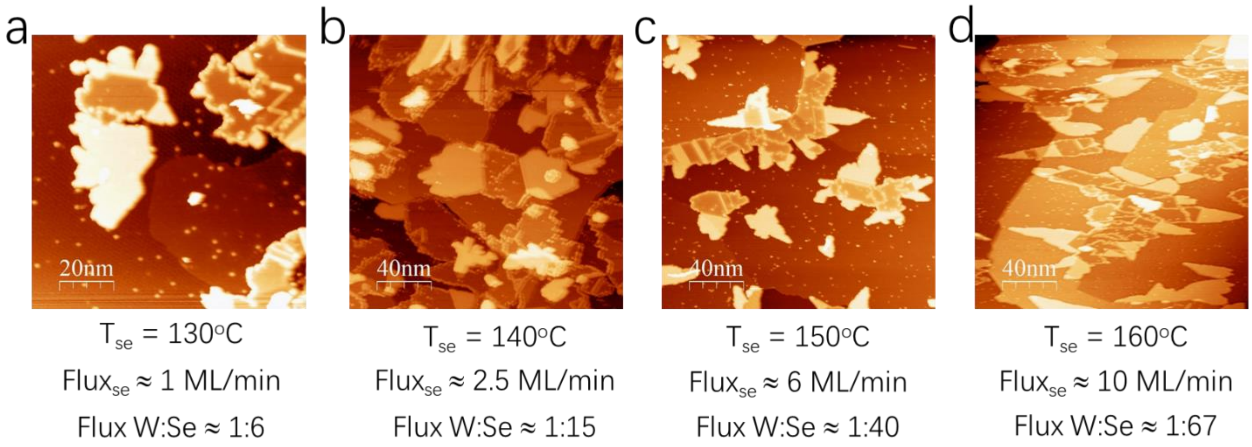

Figure S4. STM images of the WSe_2 films grown with different flux of Se.

temperature is ~250°C, which is significantly higher than the temperature of Se source and molecular beam, the Se atoms cannot be solely adsorbed on the chemically inert surface of BLG or WSe_2. Notably, the substrate temperature is lower than the temperature of W flux (> 2000°C). Therefore, the W atoms can be adsorbed on the surface of relatively cold substrate. Since the Se flux is excessive, once a W atom is adsorbed on the surface, the Se molecules will reactive with the adsorbed W atom and forms WSe_2 film. Thus $T_W > T_{sub} > T_{Se}$ growth dynamics was also reported in the MBE growth of topological insulator Bi_2Se_3 and Bi_2Te_3[1]. The growth rate of the WSe_2 film is only dominated by the flux W in such growth conditions. In Figure S4, we found the film morphology has no obviously difference.

References

1. Chen X, Ma XC, He K, Jia JF, Xue QK. Molecular beam epitaxial growth of topological insulators. *Advanced Materials* **23**, 1162-1165 (2011).
